# A Study of New Pulse Auscultation System

**DOI:** 10.3390/s150408712

**Published:** 2015-04-14

**Authors:** Ying-Yun Chen, Rong-Seng Chang

**Affiliations:** Department of Optics and Photonics, National Central University, No. 300, Jhongda Rd, Taoyuan 32001, Taiwan; E-Mail: s102286007@dop.ncu.edu.tw

**Keywords:** pulse, auscultation, condenser microphone

## Abstract

This study presents a new type of pulse auscultation system, which uses a condenser microphone to measure pulse sound waves on the wrist, captures the microphone signal for filtering, amplifies the useful signal and outputs it to an oscilloscope in analog form for waveform display and storage and delivers it to a computer to perform a Fast Fourier Transform (FFT) and convert the pulse sound waveform into a heartbeat frequency. Furthermore, it also uses an audio signal amplifier to deliver the pulse sound by speaker. The study observed the principles of Traditional Chinese Medicine’s pulsing techniques, where pulse signals at places called “cun”, “guan” and “chi” of the left hand were measured during lifting (100 g), searching (125 g) and pressing (150 g) actions. Because the system collects the vibration sound caused by the pulse, the sensor itself is not affected by the applied pressure, unlike current pulse piezoelectric sensing instruments, therefore, under any kind of pulsing pressure, it displays pulse changes and waveforms with the same accuracy. We provide an acquired pulse and waveform signal suitable for Chinese Medicine practitioners’ objective pulse diagnosis, thus providing a scientific basis for this Traditional Chinese Medicine practice. This study also presents a novel circuit design using an active filtering method. An operational amplifier with its differential features eliminates the interference from external signals, including the instant high-frequency noise. In addition, the system has the advantages of simple circuitry, cheap cost and high precision.

## 1. Introduction

Traditional Chinese Medicine’s finger pulse technique relies on finger touch, pressure sensation and vibration sensing to obtain the pulse information. This is done by applying different disturbing forces to the radial arteries by fingers, that is, the physician’s finger applies “lifting” (meaning a light pressure), “searching” (meaning mobile moderate pressure) and “pressing” (meaning heavy pressure) types of pressure on the radial artery, carefully feeling the pulse features which include but are not limited to extremity, rhythm, fluctuation, strength, thickness, hardness, smoothness and astringent information. The pulsing process can be summed up by two distinct characteristics: one is physician’s finger surface applying different degrees of pressure on the blood vessel; the other one is the physician’s finger surface sensing at the same time the tiny counter-pulsation reaction force of the pulse [1,2,3,4,5,6].

Current pulsing equipment mostly uses piezoelectric measuring methods, which have the following disadvantages: firstly, when the sensor touches the pulse via its head, the coexistence of skin tension plus the underlying soft tissue and the change of arterial axial tension as well as vascular radial pulse force have concurrent effects on the head. In the measurement of the vessel’s radial power stroke, one cannot can eliminate such tensile impacts, thus, the pulse characteristics associated with vascular tension and the axial power stroke of the pulse are indistinguishable; secondly, when the sensor presses on the measured pulse via its head, the sensor causes a deformation due to the resistance of the skin and soft tissue (*i.e.*, pulsing pressure) and the head that is pressed and touched, resulting in more measurement errors [7,8,9,10].

**Figure 1:**
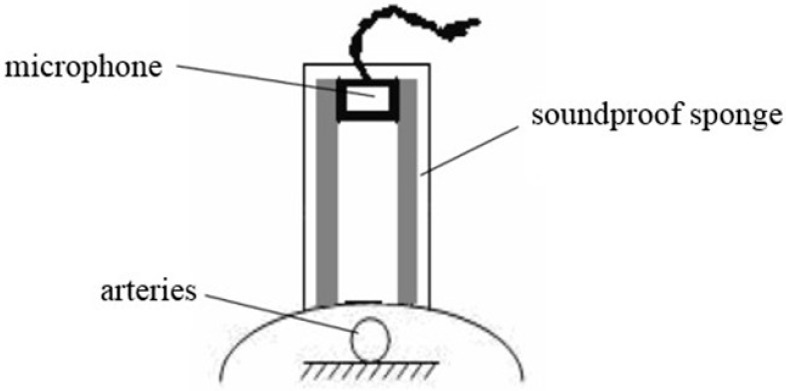
Pulse receiver.

This study presents a new pulse auscultation system (Figure 1), whose pulse receiver uses a condenser microphone as the pulse variation sensing element. To measure a signal, the microphone is pushed on the pulse measuring point through a spacer tube, and by using different pressures applied to this point, that is, the “lifting”, “searching” “and pressing” modes used in Traditional Chinese Medicine pulsing mode, different response signals are generated.

The system uses the signal captured by the pulse auscultator, which direct reflects the pulse beat. Compared to current piezoelectric sensing methods, the sensing element of the latter will display different sensitivity and accuracy depending on the pressure applied, but the system proposed herein uses the vibration noise caused by the collection of pulse strokes via a condenser microphone, so the sensor itself is not affected by any pressure and therefore, the pulse variation can be displayed with the same accuracy, regardless of which kind of pulse pressure is used [11,12]. Table 1 presents a comparison of the commercially available pulse meters and the system used here.

**Table 1:** The comparison table for the commercially available pulse meters and this system.

	Infrared Pulse Meter (Commercially Available)	Wrist Pulse Meter (Commercially Available)	This System
Sensors	Infrared emitter and detector (non-contact)	Piezoelectric sensor (contact)	Condenser microphone (non-contact)
Methods	Emits infrared to microvessels on a fingertip and receives the reflected infrared light to analyze pulse.	Places a piezoelectric sensor by the wrist, applies constant pressure and measures signal changes from pulse beats to analyze the pulse.	Uses a condenser microphone to measure and analyze the sounds generated by the pulse.
Whether it measures signal at a pulse point	No	No	Yes
System complexity	Low	High	Medium
Cost	Low	High	Medium
Whether it can measure with different pressures (based on the lifting, searching and pressing techniques used in Traditional Chinese Medicine)	No	No	Yes

## 2. New Pulse Auscultation System 

**Figure 2:**
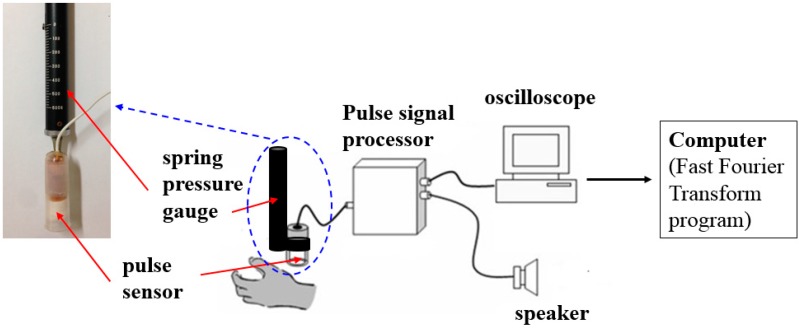
System Equipment.

The pulse measurement signal originates from the heart beat, where the diastole and systole of the heart cause the relaxation and tension of blood vessels; and upper layer of skin corresponding to the vessel will generate a clear pulse pressure therewith and the internal fluid and material inside the vessel wall will collide and rub with each other. The system is structured in three parts. The first part is the sensing transmitter; the second part is the pulse signal processor (including the zoom-in waveform of the pulse and noise filter circuits); the third part is the display and storage of waveforms and Fast Fourier Transform (FFT) operation, which mainly involves a pulse signal design for circuit acquisition, then reduces and amplifies the analog signal through low-pass filtered noise, and then displays and store the waveform via an oscilloscope, Meanwhile, it is also delivered to a computer to execute the Fast Fourier Transform (FFT) algorithm, as shown in Figure 2.

**Figure 3:**
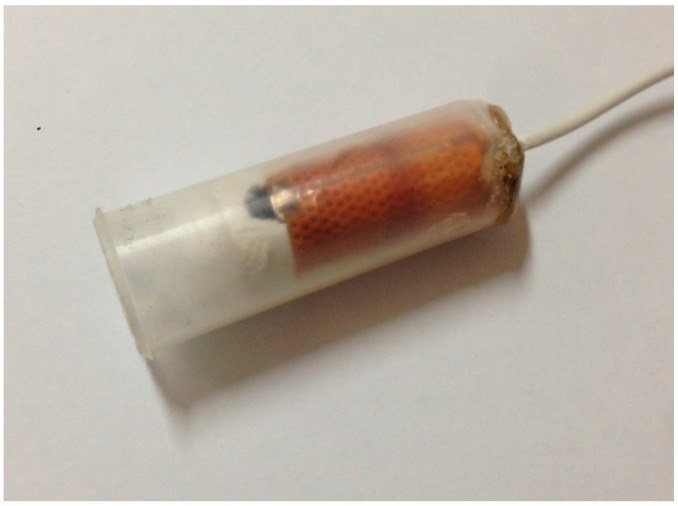
Pulse sensor.

The pulse sensor is composed of a thin plastic tube with a condenser microphone installed at the bottom, where a sponge is available for interference and noise isolation for its inner layer (Figure 3) while applying different pressures to the pulse point and thus acquiring different pulse signals. Since it is a pulse-based measurement, the output port to the lead of plastic tube is sealed, thus improving the sensitivity of the signal output from the pulse sensor.

**Figure 4:**
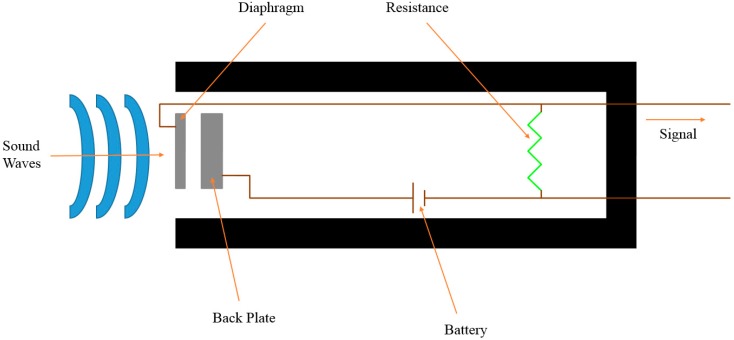
The circuit of the condenser microphone.

This study uses a condenser microphone head as shown in Figure 4 as the sensor. There are no coils or magnets, and one only needs to change the distance between the two separators in the capacitor to generate a voltage. The electret condenser microphone uses an electret material that can permanently maintain charges so there is no need to supply power to the capacitor. Sound waves enter the diaphragm of microphone to produce an audible vibration. As the substrates are stationary, the distance between the back plate and the diaphragm changes due to the vibration. According to the capacitor characteristics:

C = A/d(1)

where A is the size of the separator and d is the distance between the separators.

When the distance between the two separators changes, the capacitance C changes and then:

Q = CU(2)

where Q is the charge. The voltage of capacitor plates inside the condenser microphone remains at a fixed value. When C changes, the charge Q changes. As the condenser microphone needs to maintain a fixed plate voltage U, it requires extra power to operate. The most commonly used power source is a battery which in this study is a DC power supply. As there is an electrostatic treatment, it is in static equilibrium without electric potential and output. When the diaphragm vibrates, the distance between the two separators changes, producing an electric potential that creates a current via the resistors to make the voltage drop. The sound signals are then converted into electrical signals. As the condenser microphone offers higher sensitivity, the output is shown more precisely by a waveform [13,14]. The formula is as follows:

I = Q/T (time)(3)

V(signal) = IR(4)

The pulse signal processor is an input and output device; the input side receives a pulse signal coming from the sensor, and needs to filter the signal acquired from the microphone through a signal amplification processor, then, amplify the useful signal and output it to an oscilloscope in analog form for display and storage of the waveform. It also uses an audio signal amplifier to hear the pulse sound.

Conventional filter circuits are mainly passive. A passive filter consists of passive components and do not require power. The cutoff frequency is configured by the resistors consisting of passive components and capacitors. When used for multi-stage filtering, the circuit becomes complicated and is not able to independently perform adjustments, and it needs to do the cutoff in multiple stages. An active filter is different from the passive one in that it uses an operational amplifier as the active component. It can amplify the cutoff loss to make it constant, and therefore it needs an additional power supply. The cutoff frequency and gain are configured by the resistance and capacitance in the circuit. As the active filter has the advantages in offering better load circuit isolation and making the harmonic distortion lower than the passive filter, this circuit uses an active filter to overcome the shortcomings of the passive filter. 

**Figure 5:**
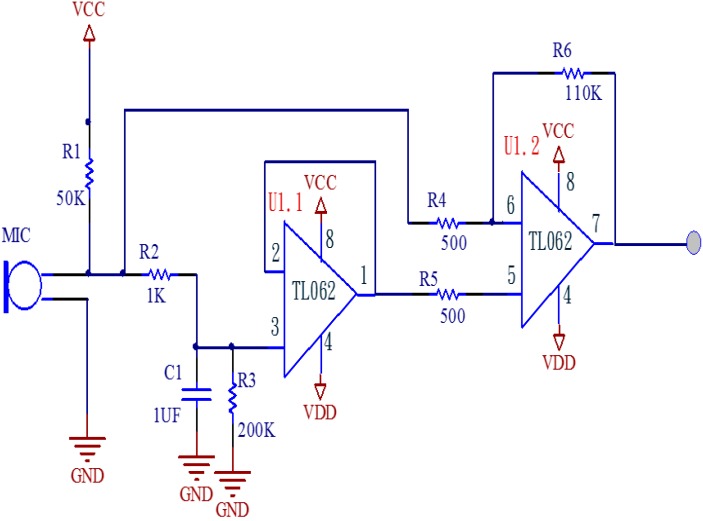
Pulse amplifying circuit.

To obtain the most realistic physiological responses, the pulse signal amplifier circuit uses a high-sensitivity condenser microphone that can detect even the smallest wave fluctuations. This study uses the model number BM-646 condenser microphone made by BSD (Taipei, Taiwan). Its specifications are: sensitivity: −70 dB ± 3 dB, impedance: 1.5 KΩ ± 30%. In the circuit design of this system, the pre-amp and power-amp both use the operational amplifier. It offers high input impedance and low output impedance, so that the actual signal output is the same as the circuit frequency. The generation of noise is random. We can use the differential characteristics of an operational amplifier to eliminate noises, including any instant high-frequency noises. A second-stage differential amplifier filters out the unwanted noises and restores and amplifies the signals as shown in Figure 5. The power consumption is rather low at 8 mA, so regular batteries can be used as the power source, preventing the interference that would be caused by regular 50 Hz/60 Hz AC power.

**Figure 6:**
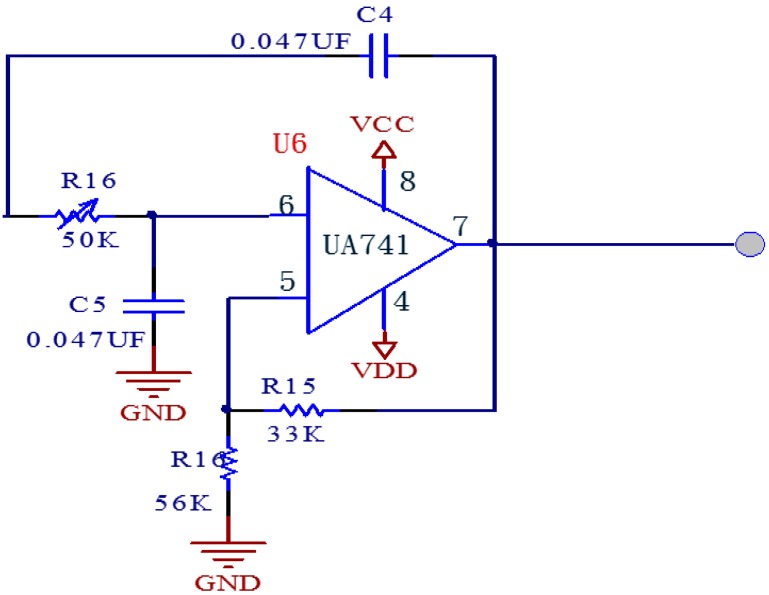
Low-pass circuit.

The circuit of Figure 6 is a low-pass circuit, designed to remove the interference of extremely low frequency noises and any interference caused by physical power on the pulse wave, further preventing aliasing, so an active second-order Sallen-Key low-pass filter was used. The operational amplifier also uses a UA741which sets the cutoff frequency at 49 Hz.

**Figure 7:**
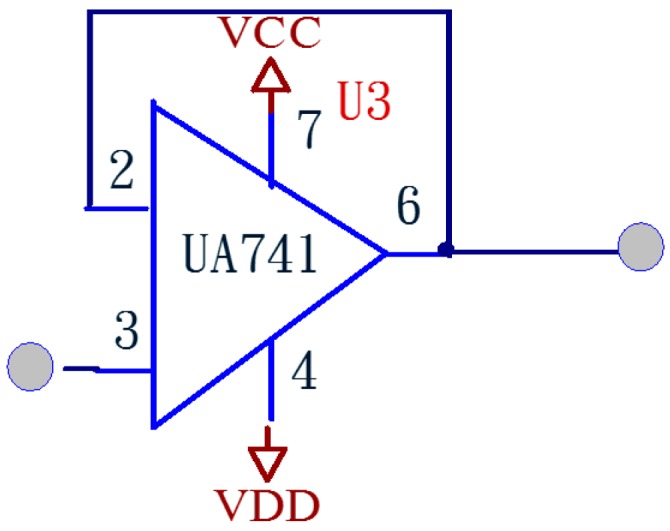
Buffer circuit.

Figure 7 shows a buffer circuit, for the sake of being compatible with the input and output impedance, high input impedes stands against low output to promote post-class speakers.

**Figure 8:**
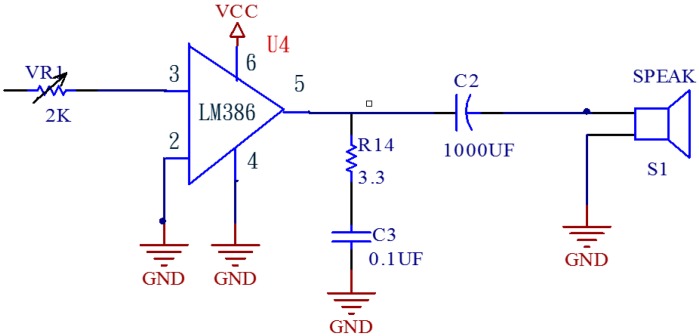
Audio amplifier.

Figure 8 shows the circuit for the audio amplifier, which uses LM 386 amplifiers; it has low power consumption with 20× internal amplification. In the case that the resistor is inserted in the 8th pin and 1st pin and capacitor is in series it can amplify by 200×, where the power is 12 V to 4 V, which makes the operating voltage very flexible; by appropriate adjustment of the VR1 value one can hear the pulse level via the speaker.

**Figure 9:**
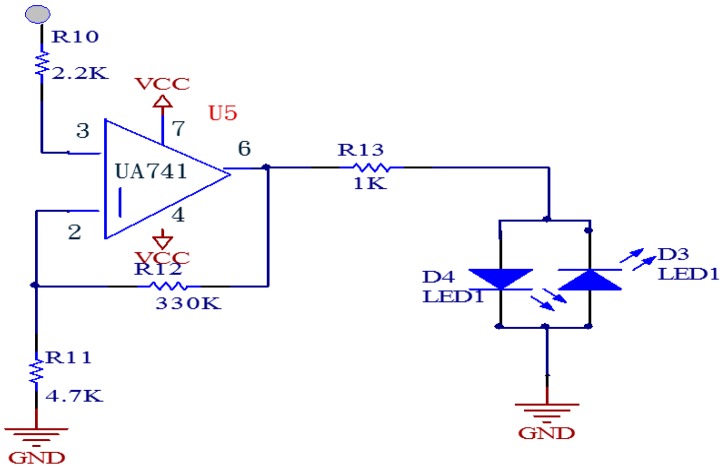
Circuit drives LED.

Figure 9 shows that in order to make the pulse stroke frequency reacted by flashing an LED, we used a UA741 operational amplifier to amplify the signal to saturation, so as to drive the LED to be functional in the saturation region; the resistance of the line in series is 1 KΩ which is a limited circuit, helping the LED to be work normally in the positive and negative half-cycle range, blinking with the pulse.

**Figure 10:**
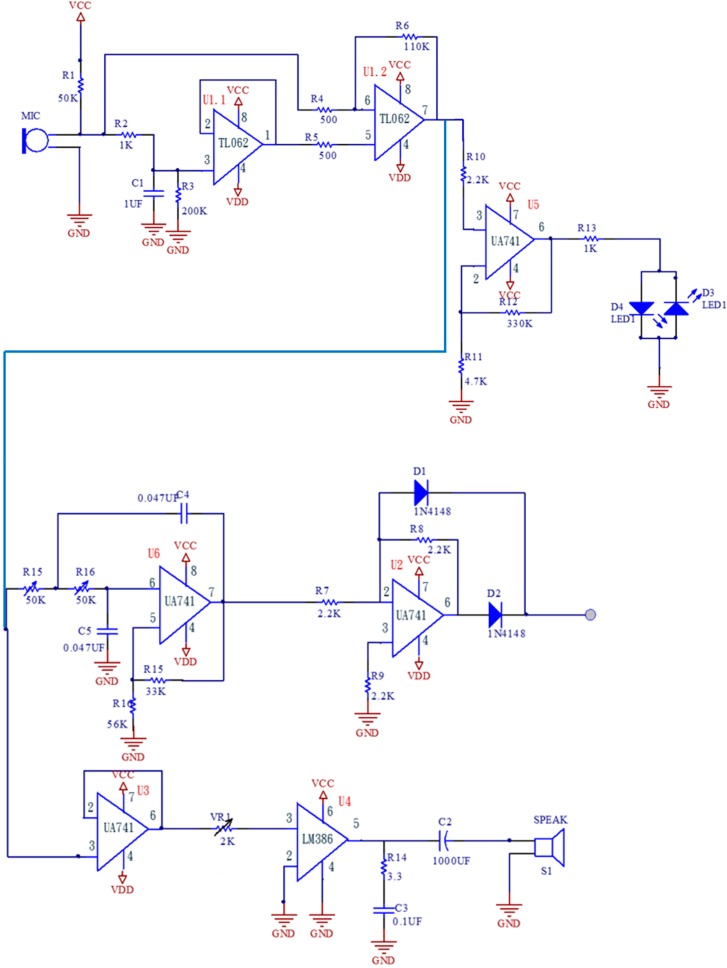
The pulse signal processor circuit.

**Figure 11:**
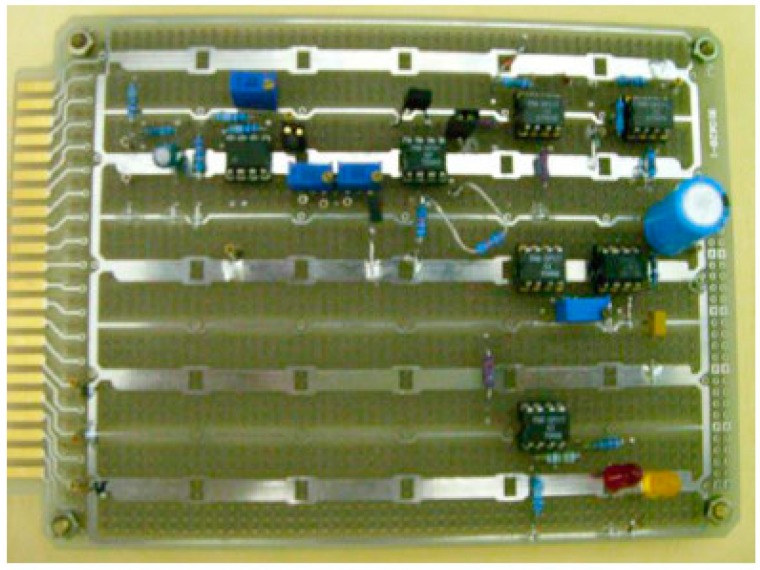
The pulse signal processor hardware.

Finally, the signal output from the pulse signal processor is an analog form of voltage (Vout as shown in the Figure 10 pulse signal processing circuit), which outputs the signal to the oscilloscope directly, and stores the pulse signal directly through oscilloscope built-in function and delivers it to a computer. Figure 11 shows the pulse signal processor hardware. We integrated the related electronic components on the same circuit board for easier measurement.

## 3. Measuring Steps

**Figure 12:**
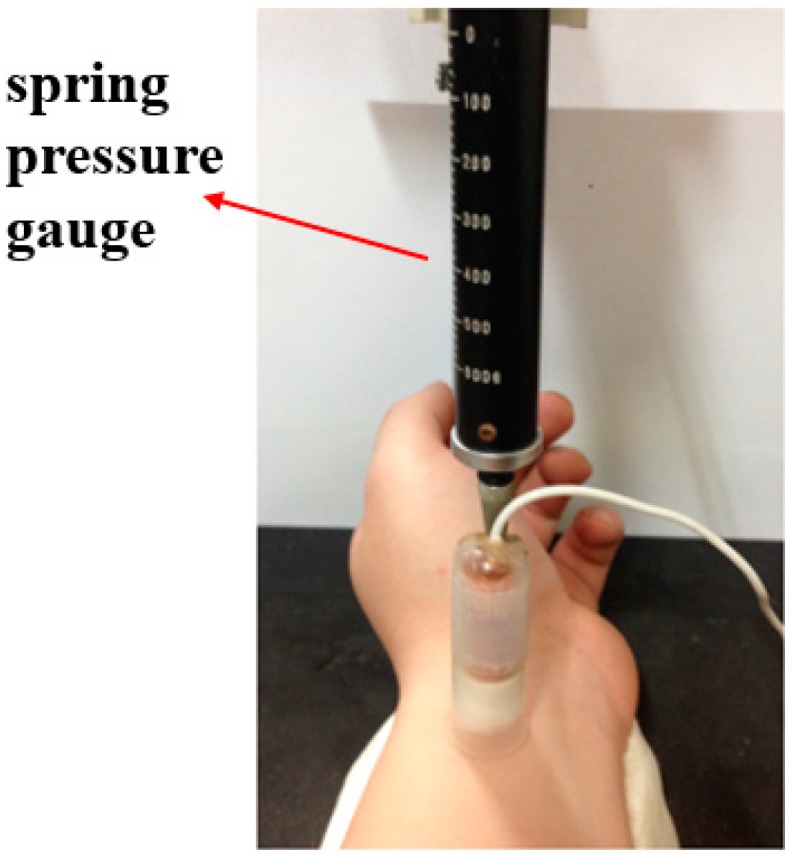
Measuring the pulse.

In order to avoid excessive variability reflected in the data acquired in the experiment, the subject must first sit and rest for five minutes. During pulsing, the subject is sitting with his wrist facing up and placed on a soft pad, with the pulse sensor on the pulses called cun, guan and chi, pressing on them with different degreess of pressure to capture pulse signals (lifting pulsing 100 g, searching pulsing 125 g and pressing pulsing 150 g). Here we set the degrees of pressure in lifting, searching, and pressing according to Yoon and Ikezono’s research [15]. They applied 100 g of absolute pressure for superficial (lifting) and 150 g for deep simulation (pressing), but they didn’t define the pressure for searching. Since the searching pressure is the average of the lifting and pressing pressure, here we define it as 125 g. When you measure it, the subject’s wrist needs to be fixed a soft pad to isolate it from any outside interference without freedom of movement, so as to reduce any impact caused by external vibration, as shown in Figure 12. The manufacturer of the spring pressure gauge is OHBA SIKI, Tokyo, Japan, and the model number is OBA600 g.

## 4. Calibration and Errors Analysis

**Figure 13:**
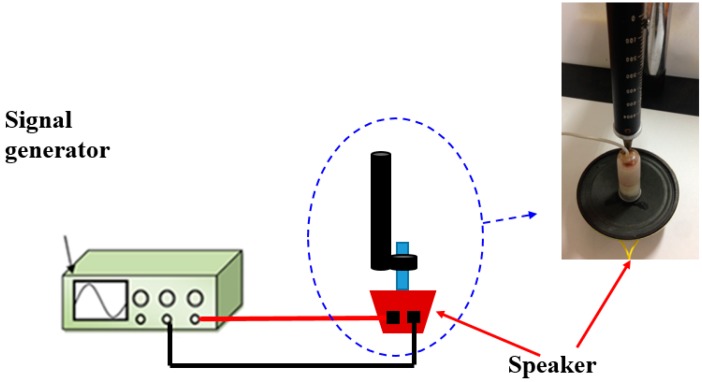
Experimental set-up of the calibration system.

**Figure 14:**
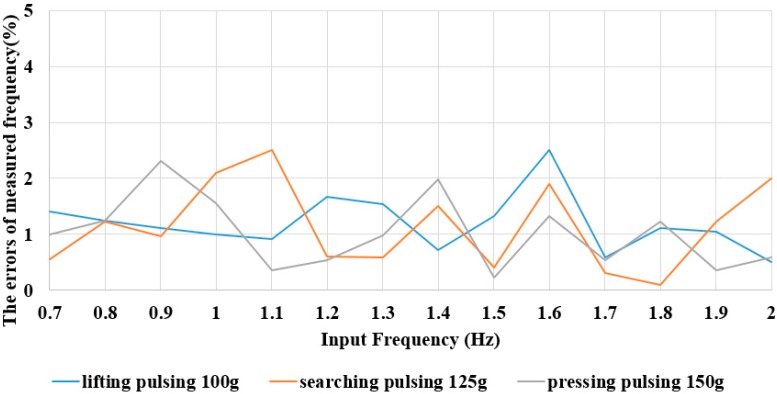
The calibrated detection system errors for 0.7 Hz–2.0 Hz frequencies.

In order to check the accuracy of the frequency measuring system for the human pulse waveform, we make a calibration: to check our measurement system, we set up a standard electric function generator to drive a speaker instead of a human wrist pulse as a standard reference target. A standard electric signal generator vibrates the speaker, and then we measure the vibrational frequency of the speaker by our method. In the next step, we compare our measurement results with the output signal of original signal generator to find the errors of our system. Figure 13 is an experimental set up for the ionystem, which is based on measuring the speaker vibration frequency driven by an electric function generator in the frequency range 0.7 Hz to 2.0 Hz under different grades of pressure (lifting pulsing 100 g, searching pulsing 125 g and pressing pulsing 150 g). The error between the measured speaker vibration frequency result by the pulse auscultation measurement frequency and the function generator frequency is less than 3%, as shown in Figure 14.

## 5. Experimental Results

**Figure 15:**
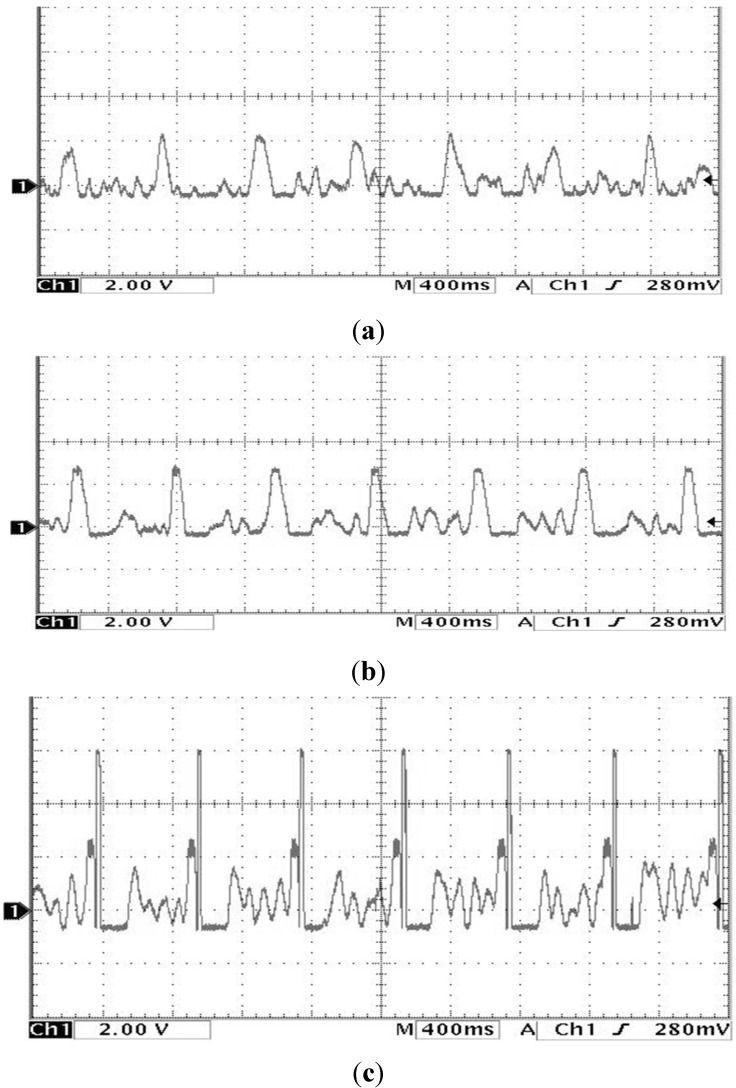
Waveform of subject A’s cun Pulse on the left hand (**a**) lifting pulsing (**b**) searching pulsing (**c**) pressing pulsing.

**Figure 16:**
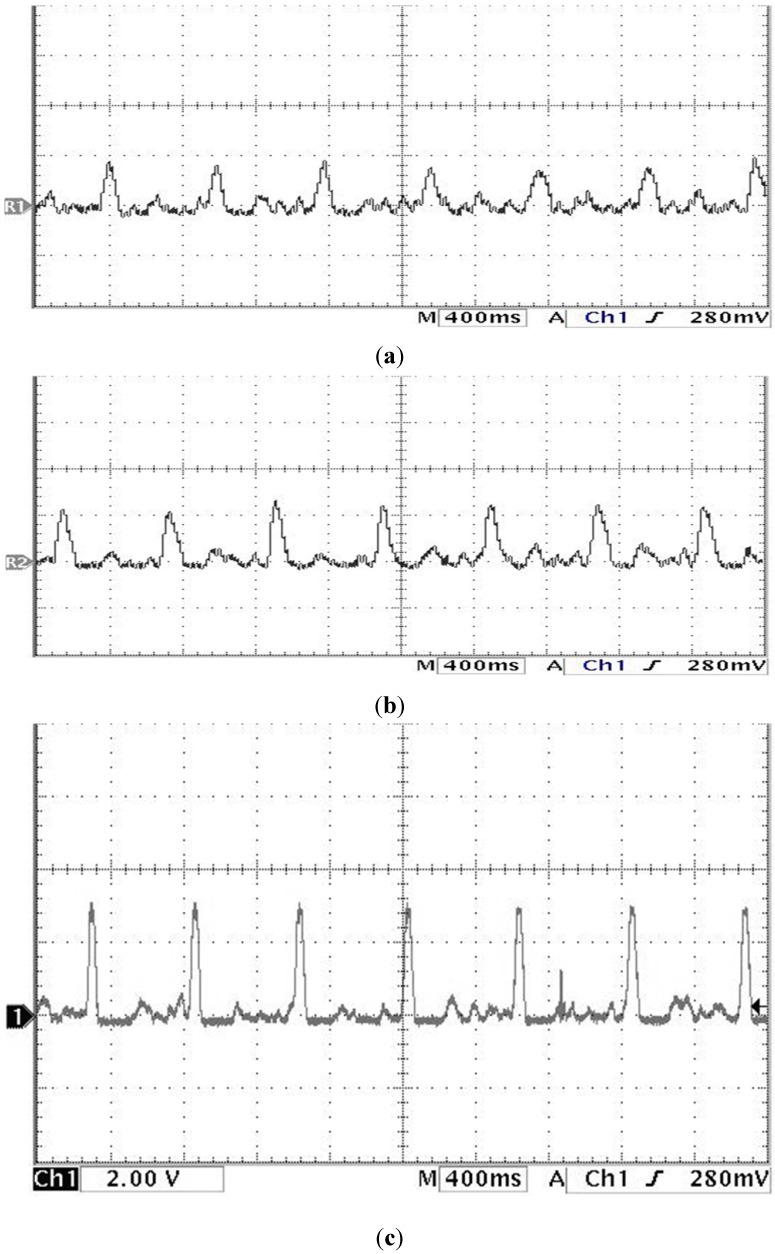
Waveform of subject A’s guan pulse on the left hand (**a**) lifting pulsing (**b**) searching pulsing (**c**) pressing pulsing.

**Figure 17:**
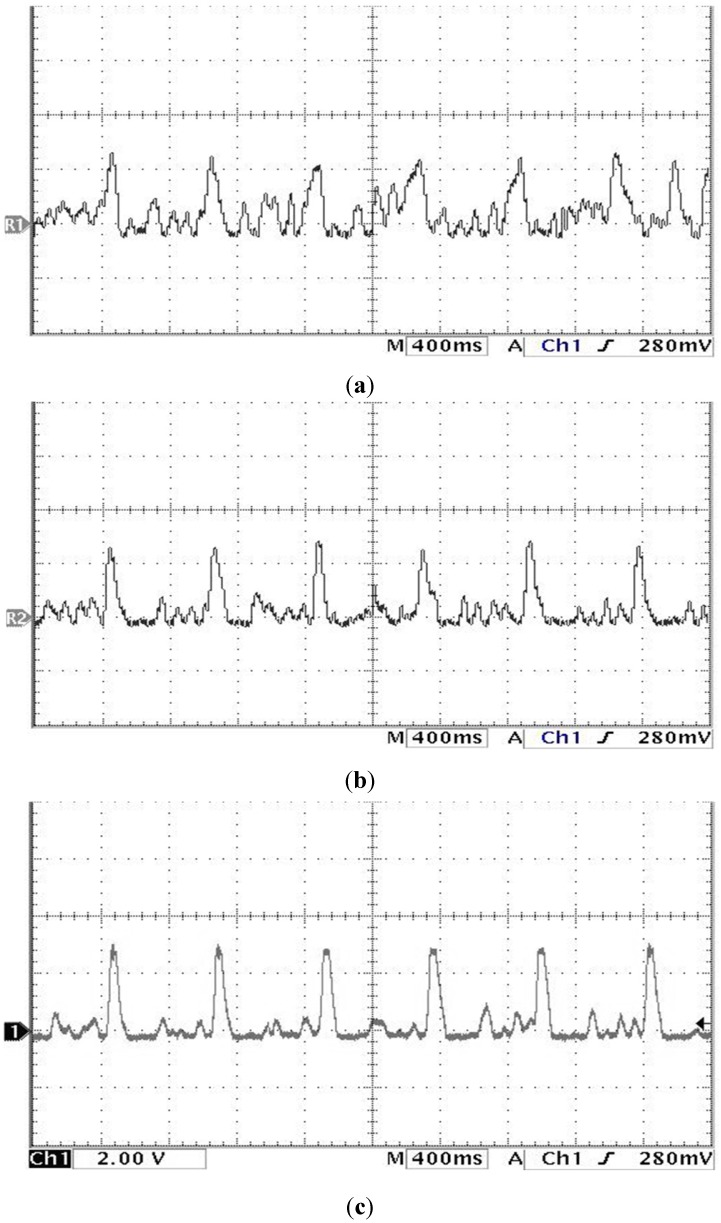
Waveform of subject A’s chi pulse on the left hand (**a**) lifting pulsing (**b**) searching pulsing (**c**) pressing pulsing.

**Figure 18:**
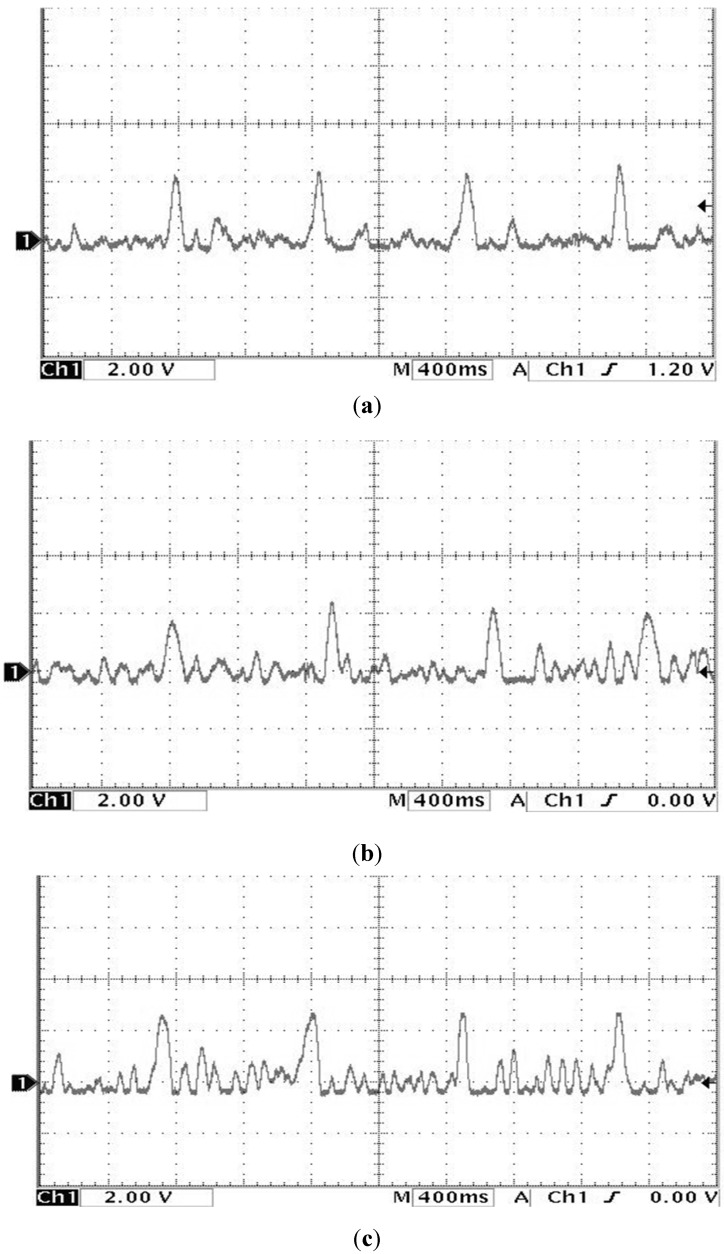
Waveform of subject B’s cun pulse on the left hand (**a**) lifting pulsing (**b**) searching pulsing (**c**) pressing pulsing.

**Figure 19:**
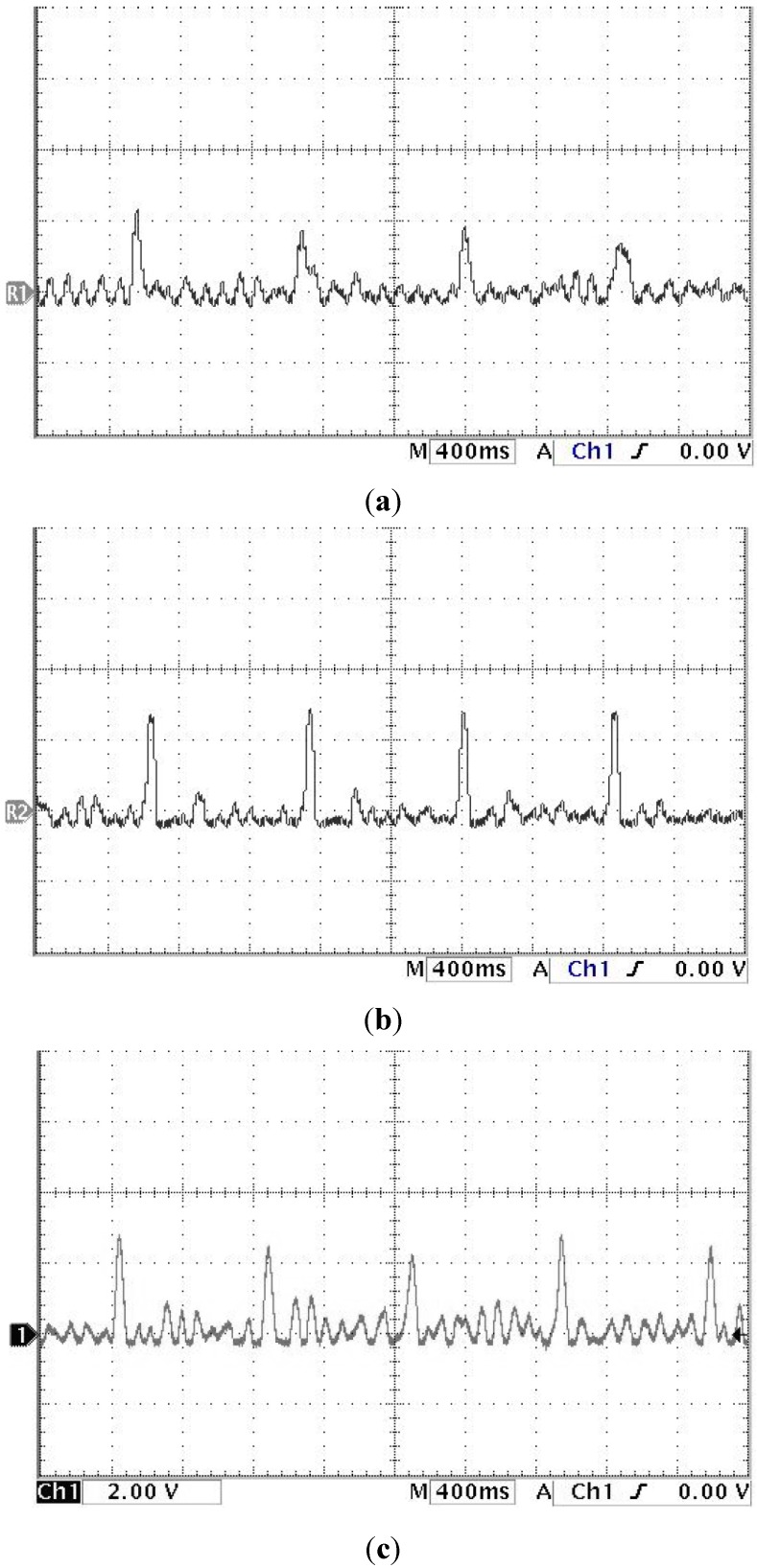
Waveform of subject B’s guan pulse on the left hand (**a**) lifting pulsing (**b**) searching pulsing (**c**) pressing pulsing.

**Figure 20:**
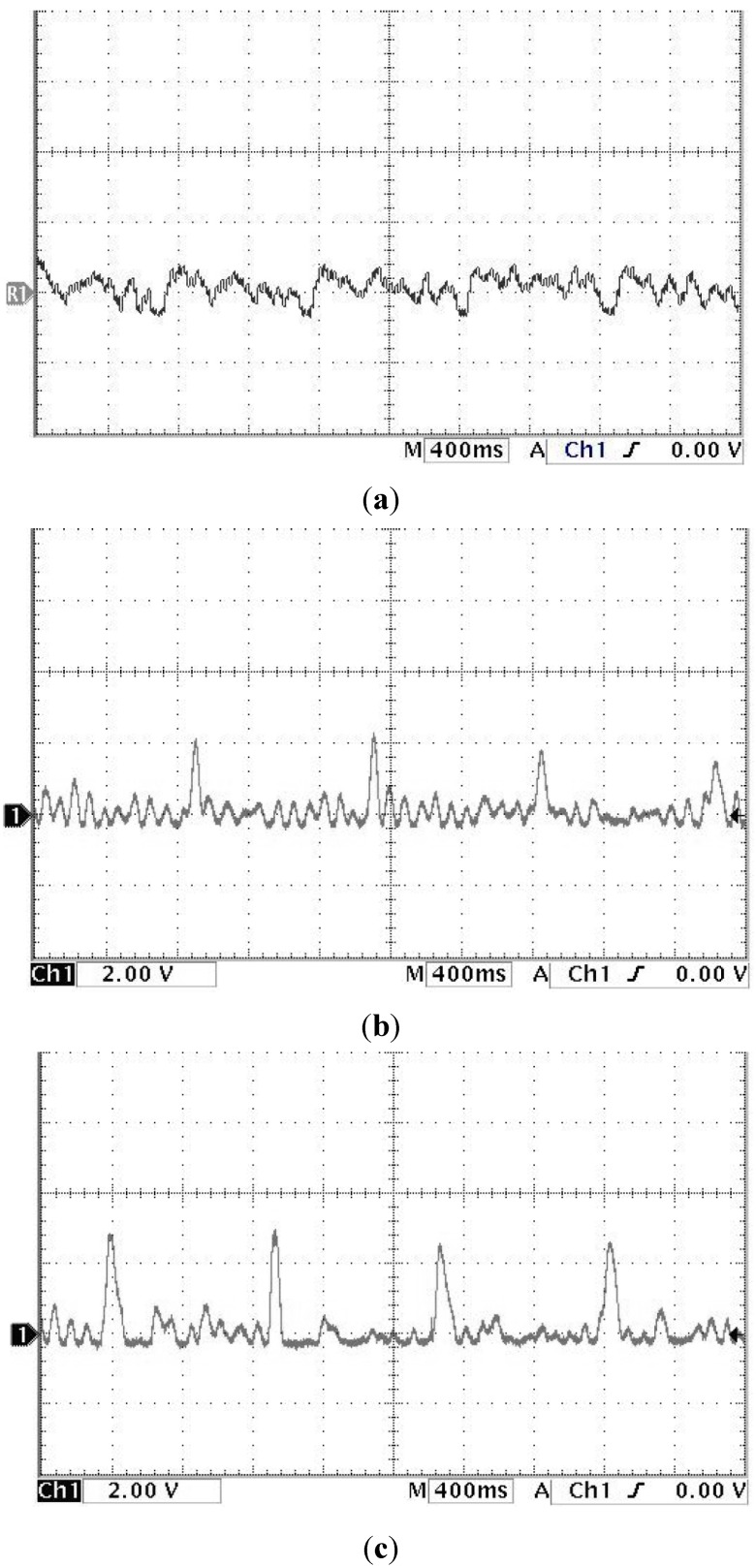
Waveform of subject B’s chi pulse on the left hand (**a**) lifting pulsing (**b**) searching pulsing (**c**) pressing pulsing.

**Figure 21:**
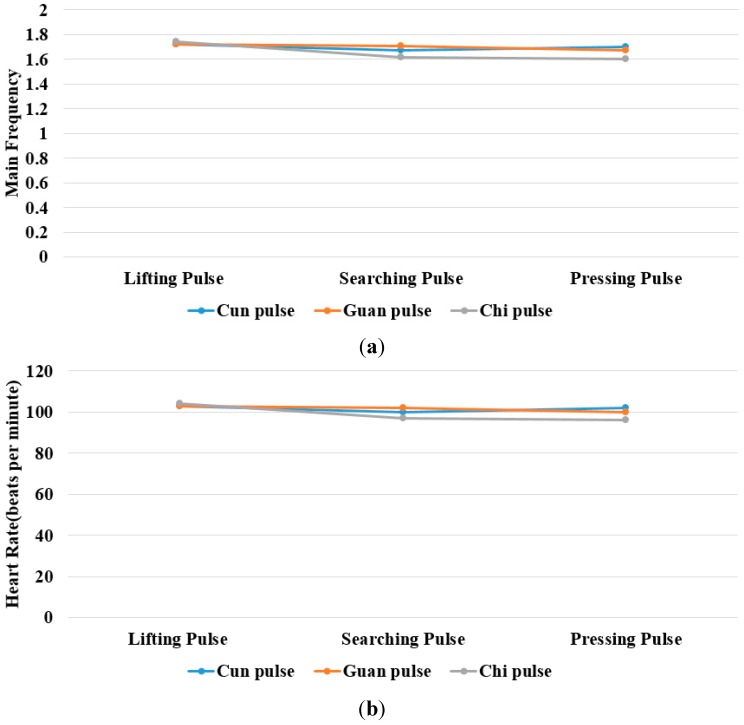
Subject A. (**a**) main frequency value; (**b**) heart Rate obtained from the sound pulse waveform Fast Fourier Transform (FFT) operation.

**Figure 22:**
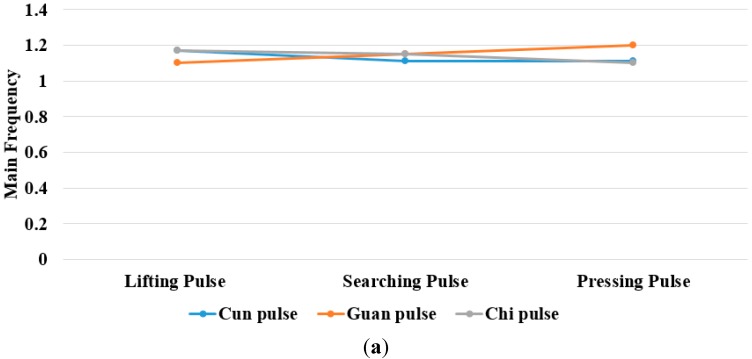

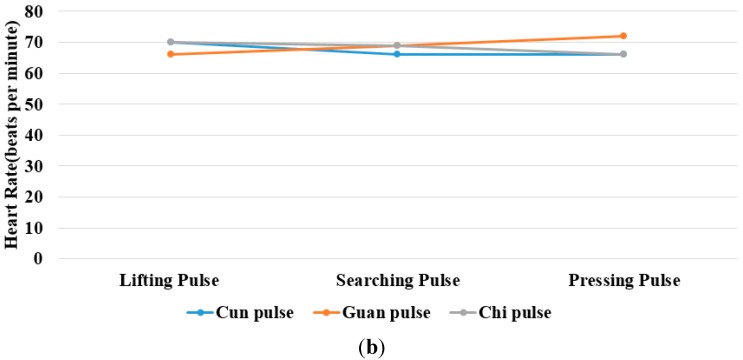
Subject B. (**a**) main frequency value; (**b**) heart rate obtained from the sound pulse waveform Fast Fourier Transform (FFT) operation.

The study showed that the heart rate of subject A is faster and subject B is quite normal by analyzing the sound pulse waveform, main frequency value and heart rate. We measured the pulses called cun, guan and chi on the left hand of subjects A and B, that is, each pulsing point is measured using the three kinds of pulsing pressure applied on these points, that is, lifting pulsing (100 g), searching pulsing (125 g) and pressing pulsing (150 g), and the sound pulse waveform results are shown in Figure 15, Figure 16, Figure 17, Figure 18, Figure 19 and Figure 20. With FFT program, the main frequency value and heart rate are as seen in Figure 21 and Figure 22.

## 6. Discussion

We verify the results shown in Figure 15, Figure 16, Figure 17, Figure 18, Figure 19 and Figure 20 with the principles of Traditional Chinese Medicine [16]. From Figure 15, Figure 16 and Figure 17, we learn that the average pulse number per breath cycle of subject A at various pressure and pulse points is about 6~7 times. As the pressure increases, the amplitude of each pulse location also increases. We consider this as a “fast pulse” based on Traditional Chinese Medicine. A fast pulse is defined as when “the pulse beats over 6 times in a cycle of breath (90~110 beats per minute), and is powerful in three pulse points under different pressure”. Also, from the results of Figure 16, Figure 17, Figure 18, Figure 19 and Figure 20, we learn that subject B is the opposite, in that the average pulse number per breath cycle is 4 times. The amplitudes of each pulse position are not much different from each other as the pressure increases. We consider this as “moderate pulse”. It is defined as “the pulse that is moderate and sluggish, beating 4 times in a cycle of breath (60–70 beats per minutes), and is weak in three pulse points under different pressure”. From the results of Figure 21 and Figure 22, we learn that the average heart rate of subject A is 100 (beats per minute), and for subject B it is 68 (beats per minute). 

The above shows the preliminary analysis we conducted for waveforms from different pulse points under different pressures. Due to our lack of enough background knowledge about Traditional Chinese Medicine pulse diagnosis, we do not discuss here and judge in medical implications of the measurements. Below we will conduct a waveform comparison. As indicated by the waveforms shown here by Subject A’s pulse (Figure 15, Figure 16 and Figure 17), when we pulsed on a single pulsing point them with different types of pulsing pressure, the lower the pressure was, the more the sub-pulse signal among each main pulse was displayed; smaller signals among main points of pulse might be inhibited by higher pressure pulsing images. In order to demonstrate our points, we measured Subject B, but with the exact opposite results coming back (Figure 18, Figure 19 and Figure 20). Therefore, different pulsing pressures on each pulse do generate different waveform and can reflect two subjects’ different physiological signs. This proves that in Traditional Chinese Medicine, doctors can distinguish the variations of pulse by applying different pulsing pressures (lifting, searching, and pressing) on the cun”, “guan”, and “chi” points.

## 7. Conclusions

We have proposed a new pulse auscultation system for scientific pulse diagnosis, which uses a condenser microphone as the sensing element together with different kinds of pulsing pressures to get varied pulse waveforms. These varied pulse waveforms are like the variety of pulse signals used in Traditional Chinese Medicine pulse diagnosis, which represent different cycle signs, and even physical conditions. Currently, we have been able to collect pulse signals under varied pulsing pressure at varied pulsing points, therefore, such a capability provides another different kind of pulse diagnosis instrument and diagnostic information. On the other hand, since each pulse waveform corresponds to different physiological signs, we shall further develop this technique with the aim of exploring signals which are beneficial for determining states of disease, and signals which is consistent with the practices of Traditional Chinese Medicine.
